# Supplementary material for: Modeling glioblastoma heterogeneity as a dynamic network of cell states
Source: Mol Syst Biol. 2021 Sep 16;17(9):e10105. doi: 10.15252/msb.202010105 (PMC8444284; doi:10.15252/msb.202010105)
Supplement: Supplementary file 6 — Source Data for Figure 5 [file MSB-17-e10105-s004.zip › Figure5A_sourcedata/GSEA_3071/nosigdb_stateII.GseaPreranked.1622795151171/RICHARDS_NOWAKOWSKI_SCIENCE_2017_OPC_UPREG.html]

Details for gene set RICHARDS\_NOWAKOWSKI\_SCIENCE\_2017\_OPC\_UPREG[GSEA]

|  || Dataset | state23071 |
| Phenotype | NoPhenotypeAvailable |
| Upregulated in class | na\_neg |
| GeneSet | RICHARDS\_NOWAKOWSKI\_SCIENCE\_2017\_OPC\_UPREG |
| Enrichment Score (ES) | -0.54981613 |
| Normalized Enrichment Score (NES) | -3.4350429 |
| Nominal p-value | 0.0 |
| FDR q-value | 0.0 |
| FWER p-Value | 0.0 |
Table: GSEA Results Summary

  

Fig 1: Enrichment plot: RICHARDS\_NOWAKOWSKI\_SCIENCE\_2017\_OPC\_UPREG      
 Profile of the Running ES Score & Positions of GeneSet Members on the Rank Ordered List

  

| PROBE | GENE SYMBOL | GENE\_TITLE | RANK IN GENE LIST | RANK METRIC SCORE | RUNNING ES | CORE ENRICHMENT || 1 | MYBL1 |  |  | 102 | 1.043 | -0.0185 | No |
| 2 | BLM |  |  | 156 | 0.942 | -0.0252 | No |
| 3 | ALCAM |  |  | 288 | 0.771 | -0.0531 | No |
| 4 | FRMD6 |  |  | 319 | 0.738 | -0.0554 | No |
| 5 | EDIL3 |  |  | 331 | 0.727 | -0.0529 | No |
| 6 | PKP4 |  |  | 444 | 0.654 | -0.0768 | No |
| 7 | NT5E |  |  | 481 | 0.637 | -0.0814 | No |
| 8 | PDLIM5 |  |  | 526 | 0.613 | -0.0882 | No |
| 9 | KLHL42 |  |  | 573 | 0.589 | -0.0957 | No |
| 10 | NTM |  |  | 592 | 0.582 | -0.0960 | No |
| 11 | EPB41L2 |  |  | 623 | 0.569 | -0.0996 | No |
| 12 | SLC25A5 |  |  | 680 | 0.537 | -0.1100 | No |
| 13 | GALNT13 |  |  | 717 | 0.527 | -0.1154 | No |
| 14 | SPCS3 |  |  | 792 | 0.501 | -0.1306 | No |
| 15 | LRP1 |  |  | 856 | 0.483 | -0.1433 | No |
| 16 | DOCK10 |  |  | 877 | 0.477 | -0.1449 | No |
| 17 | NUP50 |  |  | 888 | 0.473 | -0.1440 | No |
| 18 | DDAH1 |  |  | 1028 | 0.434 | -0.1764 | No |
| 19 | NOC3L |  |  | 1137 | 0.410 | -0.2011 | No |
| 20 | MAMLD1 |  |  | 1153 | 0.406 | -0.2020 | No |
| 21 | COL11A1 |  |  | 1163 | 0.404 | -0.2013 | No |
| 22 | STRIP2 |  |  | 1236 | 0.389 | -0.2169 | No |
| 23 | APCDD1 |  |  | 1238 | 0.389 | -0.2143 | No |
| 24 | DHCR24 |  |  | 1253 | 0.386 | -0.2151 | No |
| 25 | PCDH7 |  |  | 1329 | 0.373 | -0.2316 | No |
| 26 | NME1 |  |  | 1343 | 0.368 | -0.2322 | No |
| 27 | P2RX7 |  |  | 1383 | 0.360 | -0.2396 | No |
| 28 | ACP1 |  |  | 1396 | 0.358 | -0.2401 | No |
| 29 | TRIO |  |  | 1493 | 0.342 | -0.2622 | No |
| 30 | KHSRP |  |  | 1599 | 0.324 | -0.2867 | No |
| 31 | TMX1 |  |  | 1703 | 0.310 | -0.3108 | No |
| 32 | CCND1 |  |  | 1753 | 0.304 | -0.3211 | No |
| 33 | MTPAP |  |  | 1754 | 0.304 | -0.3189 | No |
| 34 | VCAN |  |  | 1793 | 0.299 | -0.3265 | No |
| 35 | UGDH |  |  | 1797 | 0.299 | -0.3250 | No |
| 36 | SGK1 |  |  | 1806 | 0.298 | -0.3249 | No |
| 37 | ZADH2 |  |  | 1885 | 0.287 | -0.3428 | No |
| 38 | SDC3 |  |  | 1908 | 0.284 | -0.3464 | No |
| 39 | EVI2A |  |  | 1955 | 0.278 | -0.3561 | No |
| 40 | CPT1A |  |  | 1959 | 0.278 | -0.3548 | No |
| 41 | E2F3 |  |  | 1961 | 0.277 | -0.3531 | No |
| 42 | LIMS2 |  |  | 2125 | 0.260 | -0.3929 | No |
| 43 | ARL4A |  |  | 2167 | 0.255 | -0.4016 | No |
| 44 | PTPRE |  |  | 2265 | -0.255 | -0.4246 | No |
| 45 | LRRTM3 |  |  | 2282 | -0.257 | -0.4268 | No |
| 46 | KIZ |  |  | 2301 | -0.259 | -0.4295 | No |
| 47 | TP53INP1 |  |  | 2337 | -0.263 | -0.4365 | No |
| 48 | CAT |  |  | 2344 | -0.264 | -0.4361 | No |
| 49 | OCIAD2 |  |  | 2361 | -0.266 | -0.4383 | No |
| 50 | SEMA6D |  |  | 2407 | -0.272 | -0.4478 | No |
| 51 | CANX |  |  | 2427 | -0.274 | -0.4507 | No |
| 52 | ARHGAP42 |  |  | 2449 | -0.277 | -0.4541 | No |
| 53 | OLIG2 |  |  | 2453 | -0.278 | -0.4528 | No |
| 54 | SPRY2 |  |  | 2455 | -0.278 | -0.4510 | No |
| 55 | CNP |  |  | 2457 | -0.278 | -0.4492 | No |
| 56 | ILDR2 |  |  | 2478 | -0.280 | -0.4523 | No |
| 57 | SEPT2 |  |  | 2482 | -0.281 | -0.4510 | No |
| 58 | GATM |  |  | 2501 | -0.285 | -0.4536 | No |
| 59 | STK32A |  |  | 2521 | -0.289 | -0.4563 | No |
| 60 | ATP6V0E1 |  |  | 2545 | -0.293 | -0.4601 | No |
| 61 | ABI1 |  |  | 2598 | -0.301 | -0.4712 | No |
| 62 | TMEM132B |  |  | 2623 | -0.304 | -0.4751 | No |
| 63 | PCBP4 |  |  | 2633 | -0.306 | -0.4752 | No |
| 64 | EVI5 |  |  | 2635 | -0.306 | -0.4732 | No |
| 65 | HSP90B1 |  |  | 2648 | -0.308 | -0.4740 | No |
| 66 | COPB2 |  |  | 2661 | -0.310 | -0.4748 | No |
| 67 | RAB9A |  |  | 2671 | -0.312 | -0.4749 | No |
| 68 | PRKCA |  |  | 2757 | -0.327 | -0.4943 | No |
| 69 | LEPROT |  |  | 2761 | -0.328 | -0.4926 | No |
| 70 | CFLAR |  |  | 2781 | -0.332 | -0.4951 | No |
| 71 | CPEB2 |  |  | 2787 | -0.333 | -0.4939 | No |
| 72 | PDE4B |  |  | 2788 | -0.333 | -0.4915 | No |
| 73 | ZCCHC24 |  |  | 2795 | -0.334 | -0.4906 | No |
| 74 | HIP1 |  |  | 2814 | -0.338 | -0.4927 | No |
| 75 | ATCAY |  |  | 2865 | -0.346 | -0.5030 | No |
| 76 | ST3GAL5 |  |  | 2910 | -0.358 | -0.5117 | No |
| 77 | TMX3 |  |  | 2921 | -0.359 | -0.5116 | No |
| 78 | PCDH17 |  |  | 2924 | -0.360 | -0.5095 | No |
| 79 | SLITRK2 |  |  | 2970 | -0.368 | -0.5183 | No |
| 80 | ANGPTL2 |  |  | 3019 | -0.378 | -0.5278 | No |
| 81 | APBB2 |  |  | 3023 | -0.378 | -0.5258 | No |
| 82 | ALDH6A1 |  |  | 3027 | -0.379 | -0.5238 | No |
| 83 | HIPK2 |  |  | 3029 | -0.379 | -0.5213 | No |
| 84 | PHYHIPL |  |  | 3049 | -0.385 | -0.5234 | No |
| 85 | CSGALNACT1 |  |  | 3052 | -0.386 | -0.5211 | No |
| 86 | TNK2 |  |  | 3057 | -0.387 | -0.5193 | No |
| 87 | SLC2A13 |  |  | 3064 | -0.389 | -0.5180 | No |
| 88 | LAPTM4B |  |  | 3068 | -0.389 | -0.5159 | No |
| 89 | ABAT |  |  | 3075 | -0.391 | -0.5146 | No |
| 90 | SLC22A17 |  |  | 3085 | -0.394 | -0.5140 | No |
| 91 | ZNF462 |  |  | 3086 | -0.394 | -0.5111 | No |
| 92 | SAMD8 |  |  | 3123 | -0.404 | -0.5174 | No |
| 93 | BMP7 |  |  | 3127 | -0.407 | -0.5152 | No |
| 94 | GOLIM4 |  |  | 3154 | -0.414 | -0.5188 | No |
| 95 | SETD5 |  |  | 3166 | -0.418 | -0.5186 | No |
| 96 | LAMA4 |  |  | 3193 | -0.426 | -0.5221 | No |
| 97 | SPRY4 |  |  | 3201 | -0.429 | -0.5208 | No |
| 98 | PON2 |  |  | 3216 | -0.432 | -0.5212 | No |
| 99 | TENM1 |  |  | 3235 | -0.437 | -0.5226 | No |
| 100 | FYN |  |  | 3262 | -0.444 | -0.5260 | No |
| 101 | DNM3 |  |  | 3290 | -0.454 | -0.5296 | No |
| 102 | HSPA5 |  |  | 3335 | -0.468 | -0.5375 | No |
| 103 | FAM84A |  |  | 3344 | -0.474 | -0.5361 | No |
| 104 | MARCKS |  |  | 3369 | -0.487 | -0.5386 | No |
| 105 | ZMAT3 |  |  | 3380 | -0.490 | -0.5376 | No |
| 106 | RFTN2 |  |  | 3427 | -0.511 | -0.5457 | No |
| 107 | GLCCI1 |  |  | 3444 | -0.517 | -0.5460 | Yes |
| 108 | CACNB2 |  |  | 3452 | -0.521 | -0.5440 | Yes |
| 109 | QKI |  |  | 3467 | -0.526 | -0.5437 | Yes |
| 110 | PHLDB1 |  |  | 3480 | -0.533 | -0.5429 | Yes |
| 111 | CALCRL |  |  | 3508 | -0.546 | -0.5458 | Yes |
| 112 | FABP5 |  |  | 3513 | -0.549 | -0.5428 | Yes |
| 113 | ITM2C |  |  | 3518 | -0.552 | -0.5398 | Yes |
| 114 | SKIL |  |  | 3531 | -0.560 | -0.5388 | Yes |
| 115 | WDR83OS |  |  | 3533 | -0.562 | -0.5349 | Yes |
| 116 | AASS |  |  | 3539 | -0.564 | -0.5321 | Yes |
| 117 | CTTNBP2 |  |  | 3541 | -0.565 | -0.5282 | Yes |
| 118 | OLIG1 |  |  | 3551 | -0.571 | -0.5264 | Yes |
| 119 | ERBB3 |  |  | 3561 | -0.578 | -0.5244 | Yes |
| 120 | CD9 |  |  | 3567 | -0.581 | -0.5215 | Yes |
| 121 | GNB4 |  |  | 3587 | -0.598 | -0.5220 | Yes |
| 122 | ZEB1 |  |  | 3589 | -0.598 | -0.5179 | Yes |
| 123 | DBI |  |  | 3597 | -0.601 | -0.5153 | Yes |
| 124 | SPATA6 |  |  | 3606 | -0.608 | -0.5129 | Yes |
| 125 | PHLDA1 |  |  | 3622 | -0.617 | -0.5122 | Yes |
| 126 | BAALC |  |  | 3628 | -0.625 | -0.5089 | Yes |
| 127 | CDCA7L |  |  | 3642 | -0.637 | -0.5076 | Yes |
| 128 | SHC3 |  |  | 3648 | -0.645 | -0.5041 | Yes |
| 129 | DAG1 |  |  | 3662 | -0.654 | -0.5027 | Yes |
| 130 | TNFRSF21 |  |  | 3669 | -0.660 | -0.4994 | Yes |
| 131 | LITAF |  |  | 3670 | -0.660 | -0.4946 | Yes |
| 132 | SERINC5 |  |  | 3676 | -0.664 | -0.4910 | Yes |
| 133 | PCDH9 |  |  | 3707 | -0.691 | -0.4936 | Yes |
| 134 | PLEKHH2 |  |  | 3711 | -0.693 | -0.4893 | Yes |
| 135 | LCORL |  |  | 3718 | -0.698 | -0.4858 | Yes |
| 136 | HAS2 |  |  | 3724 | -0.702 | -0.4819 | Yes |
| 137 | RAB31 |  |  | 3757 | -0.737 | -0.4847 | Yes |
| 138 | SCD5 |  |  | 3765 | -0.751 | -0.4810 | Yes |
| 139 | PLAT |  |  | 3766 | -0.752 | -0.4755 | Yes |
| 140 | SATB1 |  |  | 3776 | -0.767 | -0.4722 | Yes |
| 141 | SALL3 |  |  | 3778 | -0.768 | -0.4669 | Yes |
| 142 | GRIA2 |  |  | 3786 | -0.774 | -0.4630 | Yes |
| 143 | MAML2 |  |  | 3793 | -0.783 | -0.4588 | Yes |
| 144 | SCARB2 |  |  | 3811 | -0.798 | -0.4573 | Yes |
| 145 | WSCD1 |  |  | 3817 | -0.803 | -0.4528 | Yes |
| 146 | NLGN4X |  |  | 3823 | -0.810 | -0.4481 | Yes |
| 147 | MAGI1 |  |  | 3827 | -0.815 | -0.4429 | Yes |
| 148 | EMC10 |  |  | 3829 | -0.821 | -0.4372 | Yes |
| 149 | NLGN1 |  |  | 3836 | -0.828 | -0.4327 | Yes |
| 150 | IDH1 |  |  | 3842 | -0.832 | -0.4279 | Yes |
| 151 | LYPD1 |  |  | 3844 | -0.834 | -0.4220 | Yes |
| 152 | PCMTD2 |  |  | 3869 | -0.878 | -0.4217 | Yes |
| 153 | CHD7 |  |  | 3880 | -0.904 | -0.4177 | Yes |
| 154 | TMEFF2 |  |  | 3884 | -0.909 | -0.4118 | Yes |
| 155 | GPR155 |  |  | 3890 | -0.926 | -0.4063 | Yes |
| 156 | PROM1 |  |  | 3892 | -0.929 | -0.3998 | Yes |
| 157 | DSEL |  |  | 3897 | -0.940 | -0.3939 | Yes |
| 158 | DUSP6 |  |  | 3899 | -0.946 | -0.3873 | Yes |
| 159 | ZNF385D |  |  | 3906 | -0.959 | -0.3818 | Yes |
| 160 | CDH13 |  |  | 3908 | -0.963 | -0.3750 | Yes |
| 161 | LRRN1 |  |  | 3913 | -0.970 | -0.3690 | Yes |
| 162 | KCNQ1OT1 |  |  | 3916 | -0.988 | -0.3623 | Yes |
| 163 | SEMA5A |  |  | 3921 | -1.006 | -0.3559 | Yes |
| 164 | BCHE |  |  | 3926 | -1.012 | -0.3496 | Yes |
| 165 | ETV1 |  |  | 3934 | -1.026 | -0.3438 | Yes |
| 166 | PCDHGC3 |  |  | 3943 | -1.040 | -0.3383 | Yes |
| 167 | TRIB2 |  |  | 3944 | -1.041 | -0.3307 | Yes |
| 168 | ITPR2 |  |  | 3946 | -1.044 | -0.3233 | Yes |
| 169 | SOX2 |  |  | 3953 | -1.069 | -0.3170 | Yes |
| 170 | SOX6 |  |  | 3956 | -1.081 | -0.3096 | Yes |
| 171 | IL1RAP |  |  | 3957 | -1.082 | -0.3017 | Yes |
| 172 | ITGB8 |  |  | 3962 | -1.093 | -0.2948 | Yes |
| 173 | ETV5 |  |  | 3971 | -1.116 | -0.2887 | Yes |
| 174 | MMP16 |  |  | 3983 | -1.171 | -0.2829 | Yes |
| 175 | MYO10 |  |  | 3990 | -1.193 | -0.2757 | Yes |
| 176 | ITM2B |  |  | 3995 | -1.225 | -0.2678 | Yes |
| 177 | FGFBP3 |  |  | 3999 | -1.237 | -0.2595 | Yes |
| 178 | GPM6A |  |  | 4002 | -1.248 | -0.2509 | Yes |
| 179 | NOVA1 |  |  | 4008 | -1.312 | -0.2426 | Yes |
| 180 | SULF2 |  |  | 4023 | -1.402 | -0.2359 | Yes |
| 181 | LSAMP |  |  | 4028 | -1.442 | -0.2264 | Yes |
| 182 | MAP3K1 |  |  | 4032 | -1.477 | -0.2164 | Yes |
| 183 | SLC35F1 |  |  | 4035 | -1.515 | -0.2058 | Yes |
| 184 | SPRY1 |  |  | 4044 | -1.573 | -0.1964 | Yes |
| 185 | MAP2 |  |  | 4051 | -1.673 | -0.1857 | Yes |
| 186 | CADM4 |  |  | 4053 | -1.687 | -0.1736 | Yes |
| 187 | LIMA1 |  |  | 4056 | -1.716 | -0.1616 | Yes |
| 188 | LRRN3 |  |  | 4064 | -1.810 | -0.1501 | Yes |
| 189 | SCRG1 |  |  | 4070 | -2.021 | -0.1366 | Yes |
| 190 | PTN |  |  | 4077 | -2.187 | -0.1222 | Yes |
| 191 | S100B |  |  | 4081 | -2.383 | -0.1055 | Yes |
| 192 | PMP2 |  |  | 4082 | -2.398 | -0.0880 | Yes |
| 193 | GPM6B |  |  | 4083 | -2.435 | -0.0702 | Yes |
| 194 | C1orf61 |  |  | 4084 | -2.474 | -0.0521 | Yes |
| 195 | PDGFRA |  |  | 4096 | -3.521 | -0.0292 | Yes |
| 196 | PTPRZ1 |  |  | 4097 | -4.027 | 0.0003 | Yes |
Table: GSEA details [plain text format]

  

Fig 2: RICHARDS\_NOWAKOWSKI\_SCIENCE\_2017\_OPC\_UPREG: Random ES distribution      
 Gene set null distribution of ES for **RICHARDS\_NOWAKOWSKI\_SCIENCE\_2017\_OPC\_UPREG**
